# Quality of life and healthcare utilisation improvements after atrial fibrillation ablation

**DOI:** 10.1136/heartjnl-2020-318676

**Published:** 2021-05-05

**Authors:** Dhiraj Gupta, Johan Vijgen, Tom De Potter, Daniel Scherr, Hugo Van Herendael, Sebastien Knecht, Richard Kobza, Benjamin Berte, Niels Sandgaard, Jean-Paul Albenque, Gábor Széplaki, Yorick Stevenhagen, Philippe Taghji, Matt Wright, Mattias Duytschaever

**Affiliations:** 1 Institute of Cardiovascular Medicine and Science, Liverpool Heart and Chest Hospital, Liverpool, UK; 2 Heart Center, Jessa Hospital, Hasselt, Belgium; 3 Onze-Lieve-Vrouwziekenhuis, Aalst, Oost-Vlaanderen, Belgium; 4 Cardiology, Medical University of Graz, Graz, Austria; 5 Cardiology, Ziekenhuis Oost-Limburg, Genk, Limburg, Belgium; 6 Department of Cardiology, AZ Sint-Jan AV, Bruges, WVL, Belgium; 7 Cardiology, Luzerner Kantonsspital, Lucerne, Switzerland; 8 Department of Cardiology, Odense University Hospital, Odense, Denmark; 9 Département de Rythmologie, Clinique Pasteur, Toulouse, France; 10 Laboratory of Echocardiography, Mater Private Hospital, Dublin, Ireland; 11 Department of Cardiology, Thoraxcentrum, Enschede, The Netherlands; 12 Department of Cardiology, Clinical Clairval, Marseille, France; 13 Cardiology, Guys and St Thomas NHS Trust, London, UK

**Keywords:** atrial fibrillation, catheter ablation

## Abstract

**Objective:**

Pulmonary vein isolation (PVI) guided by a standardised CLOSE (contiguous optimised lesions) protocol has been shown to increase clinical success after catheter ablation for paroxysmal atrial fibrillation (PAF). This study analysed healthcare utilisation and quality of life (QOL) outcomes from a large multicentre prospective study, measured association between QOL and atrial fibrillation (AF) burden and identified factors associated with lack of QOL improvement.

**Methods:**

CLOSE-guided ablation was performed in 329 consecutive patients (age 61.4 years, 60.8% male) with drug-refractory PAF in 17 European centres. QOL was measured at baseline and 12 months post-ablation via Atrial Fibrillation Effect on QualiTy of Life Survey (AFEQT) and EuroQoL EQ-5D-5L questionnaires. All-cause and cardiovascular hospitalisations and cardioversions over 12 months pre-ablation and post-ablation were recorded. Rhythm monitoring included weekly and symptom-driven trans-telephonic monitoring, plus ECG and Holter monitoring at 3, 6 and 12 months. AF burden was defined as the percentage of postblanking tracings with an atrial tachyarrhythmia ≥30 s. Continuous measures across multiple time points were analysed using paired t-tests, and associations between various continuous measures were analysed using independent sample t-tests. Each statistical test used two-sided p values with a significance level of 0.05.

**Results:**

Both QOL instruments showed significant 12-month improvements across all domains: AFEQT score increased 25.1–37.5 points and 33.3%–50.8% fewer patients reporting any problem across EuroQoL EQ-5D-5L domains. Overall, AFEQT improvement was highly associated with AF burden (p=0.009 for <10% vs ≥10% burden, p<0.001 for <20% vs ≥20% burden). Cardiovascular hospitalisations were significantly decreased after ablation (42%, p=0.001). Patients without substantial improvement in AFEQT (55/301, 18.2%) had higher AFEQT and CHA_2_DS_2_-VASc scores at baseline, and higher AF burden following PVI.

**Conclusions:**

QOL improved and healthcare utilisation decreased significantly after ablation with a standardised CLOSE protocol. QOL improvement was significantly associated with impairment at baseline and AF burden after ablation.

**Trial registration number:**

NCT03062046.

## Introduction

Catheter ablation is a widely accepted treatment option for atrial fibrillation (AF). The creation of contiguous and durable lesions during pulmonary vein isolation (PVI) is essential for preventing pulmonary vein (PV) reconnection, thereby reducing the likelihood of subsequent arrhythmia recurrence.^1^ Arrhythmia recurrences can lead to repeat ablations and increased healthcare utilisation, as well as a reduction in quality of life (QOL).

Radiofrequency lesions can now be optimised with contact force (CF) sensing catheters providing real-time feedback,^2^ and utilisation of the CLOSE-PVI protocol has been shown to reduce PV reconnection rates and increase the long-term clinical success of catheter ablation for paroxysmal atrial fibrillation (PAF).^3 4^ We have recently shown via the multinational prospective VISTAX (Evaluation of Ablation Index and VISITAG™ Use for Pulmonary Vein Isolation in Patients With Paroxysmal Atrial Fibrillation) study that the CLOSE-PVI workflow is reproducible across multiple centres, with nearly 80% of patients being arrhythmia-free at 12 months postprocedure.^5^ Prospective data on healthcare utilisation and QOL after PAF ablation with this standardised workflow are limited.

The goals of this study were to report on changes in QOL and healthcare utilisation in the 12 months following catheter ablation for patients in the VISTAX study, and to investigate the relationships between QOL and measures of clinical success, including AF burden. We also sought to identify factors that were associated with lack of significant QOL improvement.

## Methods

### Study design and population

We used data collected from the VISTAX study, a prospective, non-randomised, multicentre investigation.^5^ Patients with drug-resistant symptomatic PAF were enrolled between January 2017 and March 2018 at 17 centres across 9 European countries. The research was performed without patient or public involvement.

### Ablation procedure

The CLOSE protocol, consisting of CF-guided PVI targeting an interlesion distance ≤6 mm has been previously described.^3 5^ After transseptal puncture, a left atrial three-dimensional electroanatomical map was generated using CARTO 3 System with LASSO Circular Mapping Catheter (Biosense Webster, Irvine, California, USA). Ablation was performed with either a Thermocool Smarttouch Catheter or Thermocool Smarttouch SF Catheter (Biosense Webster) to obtain a contiguous lesion set for ipsilateral circumferential PVI. VISITAG SURPOINT target values were 550 for anterior and 400 for posterior. Verification of entrance block was performed for all PVs with the LASSO catheter after a 20 min waiting period.

### Follow-up

Postprocedure, a previously ineffective anti-arrhythmic drug (AAD) therapy was permitted to be continued during the 3-month blanking period before being stopped. The use of AADs beyond the blanking period was discouraged. Patients were rigorously monitored for atrial tachyarrhythmia recurrences via weekly and symptom-driven trans-telephonic (TTM) monitoring, as well as 12-lead ECG and 24-hour Holter monitoring at 3, 6 and 12 months postprocedure. Management of recurrences, including repeat ablation(s) during follow-up was per investigator discretion.

### Assessment of quality of life and healthcare utilisation

#### Quality of life

QOL was evaluated at baseline and at the 12-month postprocedure clinic visit. Patients completed two self-administered questionnaires—the Atrial Fibrillation Effect on QualiTy of Life Survey (AFEQT) and the EuroQoL EQ-5D-5L.

The AFEQT is a validated, 20-question disease-specific survey designed to measure patients’ perceptions of their AF symptoms, functional impairment, treatment concerns and satisfaction with treatment.^6–8^ Each question has 7-point Likert responses ranging from the most severe limitations or symptoms to none. The survey produces an overall score and subscale scores for each of three domains: symptoms, daily activities and treatment concerns, with two satisfaction questions scored individually. Each score is normalised to a range of 0–100, with higher scores indicating higher QOL.^9^ An increase of 5 points in the overall AFEQT score has been identified as a clinically important difference based on analysis of 1347 patients in an AF registry.^9^

The EQ-5D-5L is a simple validated questionnaire used to assess broad health-related QOL in diverse patient populations.^10 11^ One component is a 5-dimension health status comprising 5-level responses to questions on mobility, self-care, usual activities, pain/discomfort and anxiety/depression. The second component is a visual analogue scale (EQ-VAS) for self-evaluation of overall health status on a 20 cm vertical line.

#### Atrial tachyarrhythmia recurrence and burden

An atrial tachyarrhythmia recurrence was defined as any device-recorded episode of AF, atrial tachycardia (AT) or atrial flutter (AFL) lasting ≥30 s. To evaluate the relationship between postblanking atrial tachyarrhythmia burden and QOL, burden was defined as the percentage of days beyond the 90-day blanking period with at least one device-recorded AF/AT/AFL episode. The denominator used for this percentage was the number of days after the 90-day blanking period that a patient had a tracing. The device recordings used to create this burden end point included core-lab confirmed outcomes from TTM, ECG and Holter monitors, along with AE and postprocedure arrhythmia logs. Overlap among these records was not a concern due to counting each day only once, either as a day with or without burden. For comparisons of patients with higher versus lower burden, cohorts were created by using two cutoffs: burden of <10% vs ≥10% and<20% vs ≥20%.

#### Healthcare utilisation: hospitalisation, cardioversion and repeat ablation

All-cause hospitalisation, cardiovascular hospitalisation and direct current cardioversion were assessed by comparing 12-month pre-ablation vs 12-month post-ablation rates. Hospitalisations were defined as visits with at least an overnight stay, not concurrent with the ablation procedure. These were further classified as cardiovascular hospitalisations or non-cardiovascular hospitalisations. Repeat ablations were also captured separately without the requirement of an overnight stay.

### Statistical methods

Changes in overall AFEQT scores and EQ-VAS scores from baseline to 12 months were summarised for patients who completed questionnaires at both time points. Paired t-tests were used to compare continuous measures across multiple time points, including QOL scores at baseline vs 12 months, and disease-related healthcare utilisations in the 12 months preprocedure versus postprocedure. Independent sample t-tests were used to measure associations between different continuous measures, including average change in overall AFEQT score and clinical success outcomes. McNemar tests were used to compare nominal outcomes, including EQ-5D-5L domains at baseline vs 12 months and the number of patients with at least one all-cause hospitalisation, cardiovascular hospitalisation and cardioversion in the 12-month periods preprocedure versus postprocedure. All statistical tests used two-sided p values and a significance level of 0.05.

Linear regression was used to determine which factors had the greatest impact on a patient’s level of QOL improvement, thus providing insight as to why some patients did not show meaningful gains. One model explored baseline AFEQT scores, patient characteristics and medical history as potential explanatory variables. A separate model explored the impact of post-ablation outcomes, including healthcare utilisation and residual AF burden within 12 months post-ablation. All variables with p values of ≤0.15 in univariate models were tested for inclusion in the multivariable models. Stepwise selection was then used to retain only variables that were significant at a level of 0.10 in the final models.

All statistical analyses were performed using SAS V.9.4 (SAS Institute, Cary, North Carolina, USA).

## Results

### Patient population

A total of 340 patients were enrolled in the study, with 329 meeting all eligibility criteria, including a CLOSE ablation procedure, as required for the evaluable population. The mean age was 61.4 years and 60.8% were male. Baseline patient characteristics are summarised in table 1.

**Table 1:** Baseline patient characteristics

Characteristics	Evaluable population (n=329)
Age, years	61.4±10.0
Male	200/329 (60.8)
Body mass index	27.6±4.2
Left ventricular ejection fraction, %	61.6±6.9
Left atrial diameter, mm	39.1±5.2
CHA_2_DS_2_-VASc score	1.6±1.4
**Patient medical history**	
Atrial fibrillation duration, months	22.0 (6.0, 60.0)
Atrial flutter	63/329 (19.1)
Hypertension	138/329 (41.9)
Type II diabetes	22/329 (6.7)
Coronary disease	34/329 (10.3)
Thromboembolic events	19/329 (5.8)
Congestive heart failure	9/329 (2.7)
NYHA class I	4/329 (1.2)
NYHA class II	5/329 (1.5)
**Previously failed anti-arrhythmic drug(s)**	
Number of failed anti-arrhythmic drugs	1.7±0.7
Failed any class I/III anti-arrhythmic drug	242/329 (73.6)
Failed any class II/IV anti-arrhythmic drug	232/329 (70.5)

Numbers in the table are represented as mean±SD, n/N (%) or median (IQR).

NYHA, New York Heart Association.

### Quality of life

**Figure 1:**
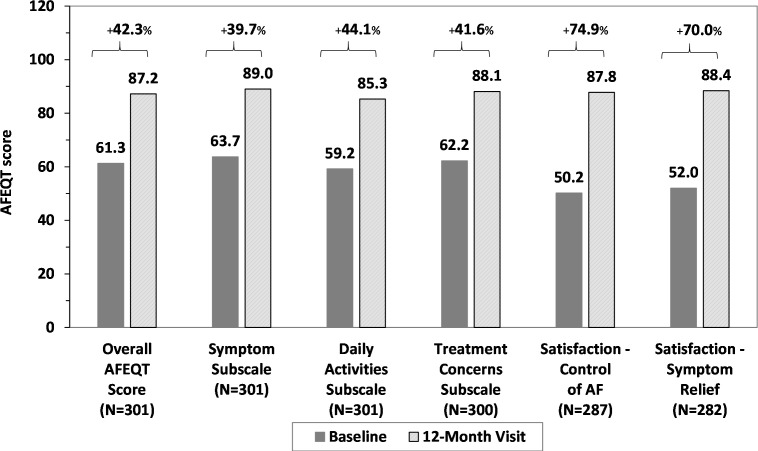
Change in mean Atrial Fibrillation Effect on QualiTy of Life Survey (AFEQT) scores from baseline to 12 months postprocedure. All score improvements are statistically significant (p<0.001). AF, atrial fibrillation.

Of the 329 patients in the evaluable population, 301 (91.5%) had AFEQT questionnaires completed at both baseline and 12 months. The mean overall AFEQT score at baseline was 61.3±20.3, and this increased significantly by 25.7±21.3 points to 87.2±15.2 at 12 months (p<0.001). An increase in AFEQT scores was also observed across each domain and satisfaction question (p<0.001 for each) (figure 1).

**Figure 2:**
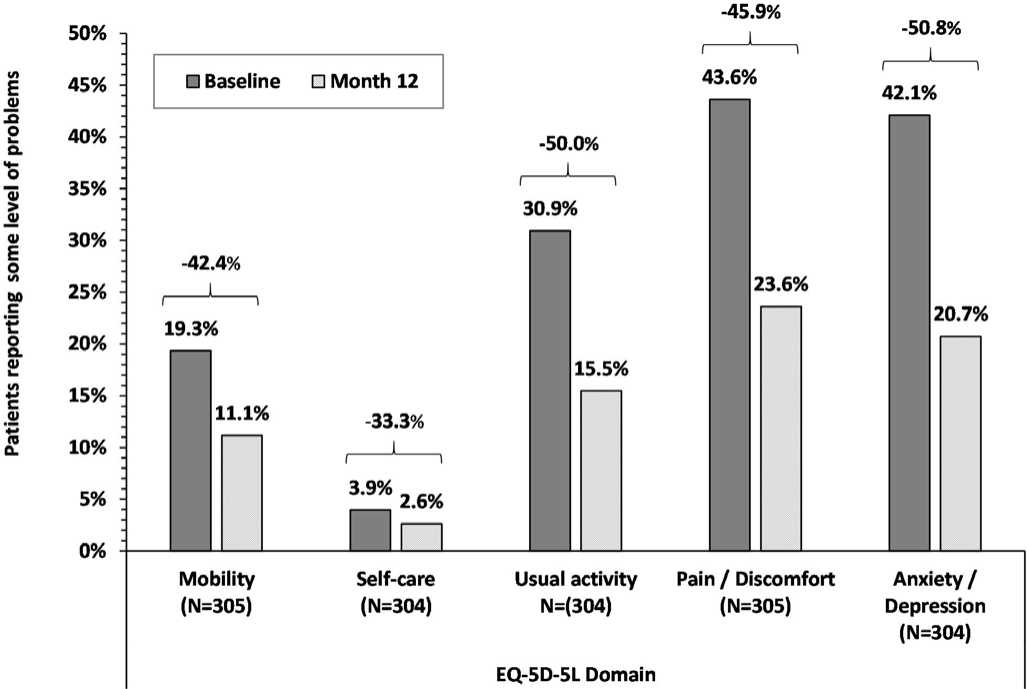
EuroQoL EQ-5D-5L change in percentage of patients with problems. Patients with problems include those reporting ‘slight’ or worse level of problem versus ‘none’. All decreases were statistically significant (p<0.001) except the self-care domain.

Consistent with AFEQT improvement, the mean EQ-VAS score increased by 8.7±14.6 points from 72.7±15.1 at baseline to 81.4±13.8 at 12 months post-ablation (p<0.001). In addition, fewer patients reported having problems in each domain of the EQ-5D-5L descriptive questionnaire at 12 months postprocedure relative to baseline, with decreases ranging from 33.3% to 50.8% across the five domains (figure 2). These decreases were statistically significant (p<0.001) for all domains except self-care.

### Symptomatic atrial tachyarrhythmia recurrence and residual burden

**Figure 3:**
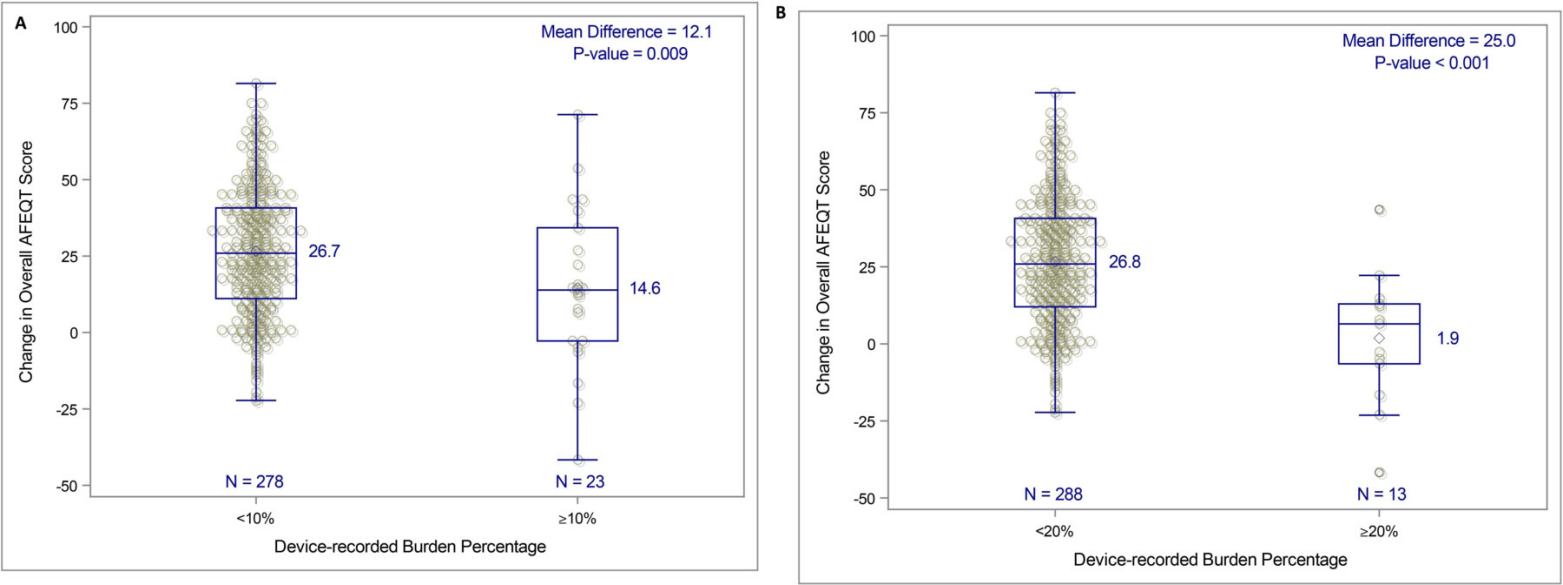
Change from baseline in 12-month overall Atrial Fibrillation Effect on QualiTy of Life Survey (AFEQT) score by atrial fibrillation (AF) burden level. (A) Change in overall AFEQT score by residual AF burden <10% or ≥10%. (B) Change in overall AFEQT score by residual AF burden <10% or ≥20% AF burden is defined as the percentage of recording days with an episode of atrial fibrillation, atrial tachycardia and/or atrial flutter lasting ≥30 s.

**Figure 4:**
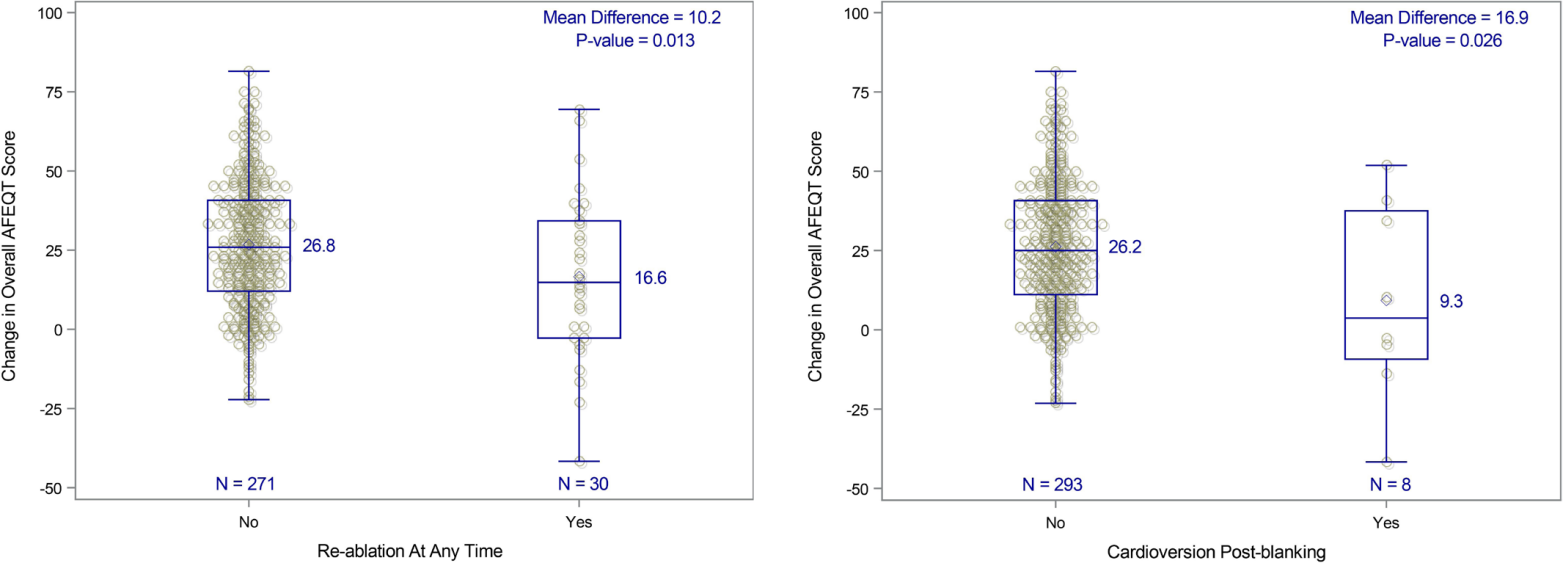
Changes in quality of life by post-ablation healthcare utilisation. (A) Change in overall Atrial Fibrillation Effect on QualiTy of Life Survey (AFEQT) score by re-ablation status. (B) Change in overall AFEQT score by cardioversion postblanking.

Kaplan-Meier estimated 12-month survival from documented symptomatic atrial tachyarrhythmia recurrence was 82.9% (95% CI 78.8% to 87.0%). Improvement in QOL was significantly associated with residual atrial tachyarrhythmia burden. The mean increase in the overall AFEQT score was higher in patients with <10% burden vs ≥10% (12 points difference, p=0.009) and with further differentiation of patients with <20% burden vs ≥20% (25.0 points difference, p<0.001) (figure 3). Greater improvements in AFEQT scores were also seen in patients who did not require repeat ablation versus those who did (figure 4A), and in patients who did not require post-blanking period cardioversion (figure 4B).

### Factors influencing level of improvement in QOL

Using the commonly accepted threshold of ≥5 points increase in overall AFEQT score as reflective of a clinically meaningful improvement in QOL, 246 (81.7%) patients exceeded this threshold while 55 (18.3%) did not. At baseline, the two groups showed no differences in PAF duration, number of failed AADs or presence of coexisting AFL. However, the patients who failed to achieve a clinically meaningful improvement had higher mean overall AFEQT scores at baseline (78.0 vs 57.7, p<0.001), with 52.7% vs 11.4% starting at scores of >80. These patients also had significantly higher CHA_2_DS_2_-VASc scores (1.9 vs 1.5, p=0.027) and more required cardioversion within 12 months prior to their ablation (30.9% vs 12.2%, p<0.001).

After ablation, patients without clinically significant improvement in QOL had borderline significantly higher mean residual AF burden (6.5% vs 2.0%, p=0.051), and were almost four times as likely to have had a burden level of >20% (10.9% vs 2.8%, p=0.008). In addition, more of these patients tended to have documented arrhythmia recurrence (30.9% vs 19.1%, p=0.053) and more required repeat ablation (18.2% vs 8.1%, p=0.024). They also had higher, but not statistically significant, rates of hospitalisation and cardioversion. All comparisons of baseline characteristics and post-ablation outcomes are shown in table 2.

**Table 2:** Differences between patients with and without significant QOL gains

Characteristics and outcomes	AFEQT improvement		P value
	≥5 points (n=246)	<5 points (n=55)	
**Overall AFEQT score**			
Baseline score	57.7±18.4	78.0±19.8	<0.001
High score (>80) at baseline	28 (11.4)	29 (52.7)	<0.001
12-month score	90.2±11.9	73.6±20.9	<0.001
Change in score—baseline to 12 months	32.5±17.0	−4.4±8.9	<0.001
**Characteristics**			
Age, years	61.0±10.1	62.9±9.4	0.194
Male	156 (63.4)	33 (60.0)	0.636
Body mass index	27.4±4.2	28.2±4.5	0.226
Left ventricular ejection fraction, %	61.5±6.7	61.2±7.3	0.768
Left atrial diameter, mm	39.0±5.3	39.2±5.1	0.845
CHA_2_DS_2_-VASc score	1.5±1.4	1.9±1.4	0.027
**Patient medical history**			
Atrial fibrillation duration, months	23.5 (6.0, 60.0)	20.0 (8.0, 72.0)	0.652
Atrial flutter	48 (19.5)	11 (20.0)	0.934
Hypertension	96 (39.0)	32 (58.2)	0.009
Type II diabetes	15 (6.1)	6 (10.9)	0.205
Coronary disease	21 (8.5)	8 (14.5)	0.172
Thromboembolic events	14 (5.7)	5 (9.1)	0.349
Congestive heart failure (NYHA class I/II)	6 (2.4)	3 (5.5)	0.235
**Utilisation within 12 months pre-ablation**			
All-cause hospitalisation	58 (23.6)	15 (27.3)	0.563
Cardiovascular hospitalisation	55 (22.4)	15 (27.3)	0.435
Cardioversion	30 (12.2)	17 (30.9)	<0.001
**Previously failed anti-arrhythmic drug(s**)			
Number of failed anti-arrhythmic drugs	1.6±0.8	1.7±0.7	0.666
Failed any class I/III anti-arrhythmic drug	173 (70.3)	45 (81.8)	0.085
**Outcomes within 12 months post-ablation**			
Recurrence after 90-day blanking	47 (19.1)	17 (30.9)	0.053
Atrial tachyarrhythmia burden	2.0%±6.7%	6.5%±16.5%	0.051
Burden of ≥10%	16 (6.5)	7 (12.7)	0.116
Burden of ≥20%	7 (2.8)	6 (10.9)	0.008
Repeat ablation	20 (8.1)	10 (18.2)	0.024
All-cause hospitalisation	46 (18.7)	14 (25.5)	0.257
Cardiovascular hospitalisation	30 (12.2)	11 (20.0)	0.127
Cardioversion	14 (5.7)	5 (9.1)	0.349

Numbers in the table are represented as mean±SD, n (%) or median (IQR).

P values based on t-test for means, Kruskal-Wallis test for medians and χ^2^ test for counts.

AFEQT, Atrial Fibrillation Effect onQualiTy of Life Survey; NYHA, New York Heart Association; QOL, quality of life.

**Figure 5:**
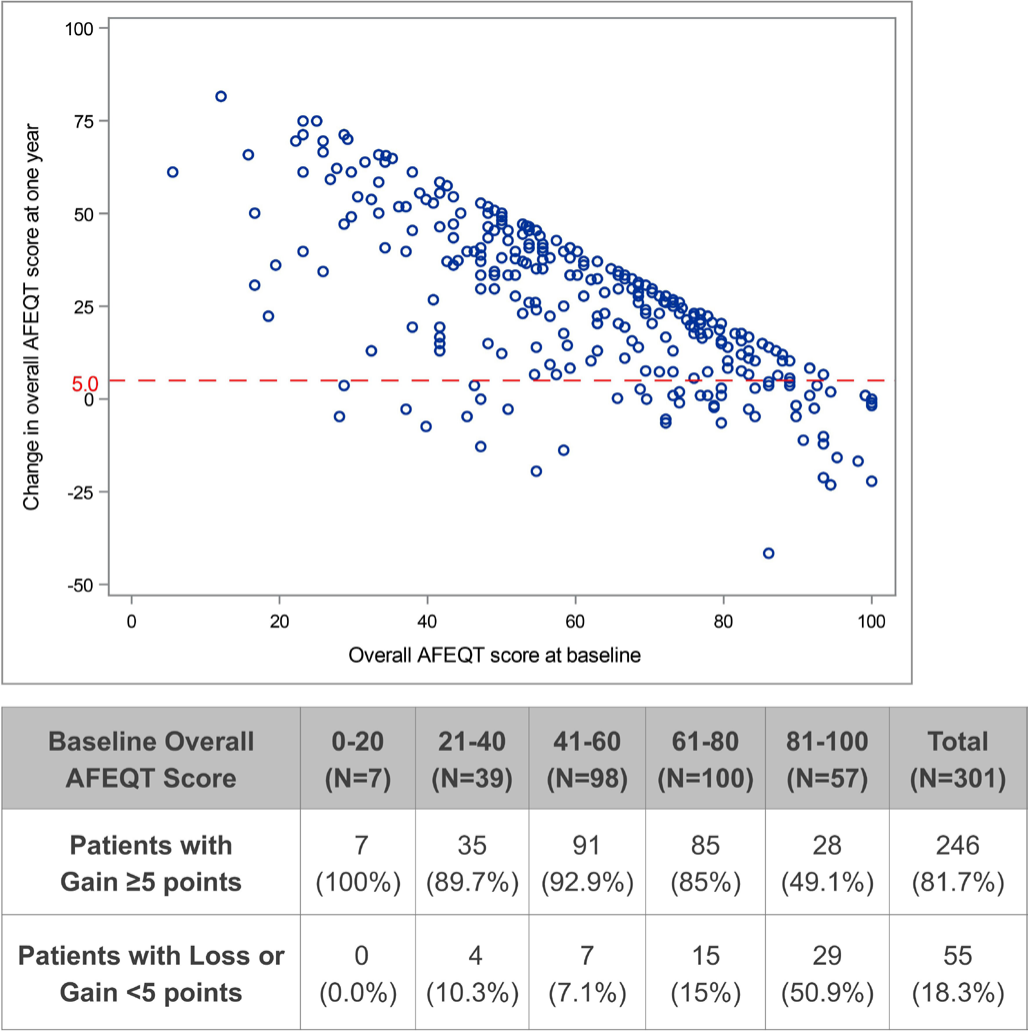
Atrial Fibrillation Effect on Quality of Life Survey (AFEQT) change versus baseline score.

A scatter plot of the changes from baseline in overall AFEQT score at the 12-month visit versus the baseline scores shows a clear trend of higher improvement in patients with lower QOL at baseline (figure 5). Reductions in the potential level of improvement for patients with high baseline QOL resulted in fewer of these patients experiencing significant levels of improvement compared with patients with moderate or low baseline QOL.

The multivariable linear regression analysis showed that baseline AFEQT score was the most significant baseline predictor of improvement in the overall score at 12 months (p<0.001). Every 10-point increase in the baseline overall AFEQT score was associated with a mean 7.7-point reduction in score improvement at 1 year (95% CI 6.9 to 8.5, p<0.001). CHA_2_DS_2_-VASc score was the only additional significant predictor in the baseline explanatory model (p=0.004). Residual atrial tachyarrhythmia burden was the most significant post-ablation predictor of change in QOL (p<0.001), and no additional post-ablation predictors were significant after adjusting for burden in the post-ablation explanatory model.

### Healthcare utilisation: hospitalisation, cardioversion and repeat ablation

**Figure 6:**
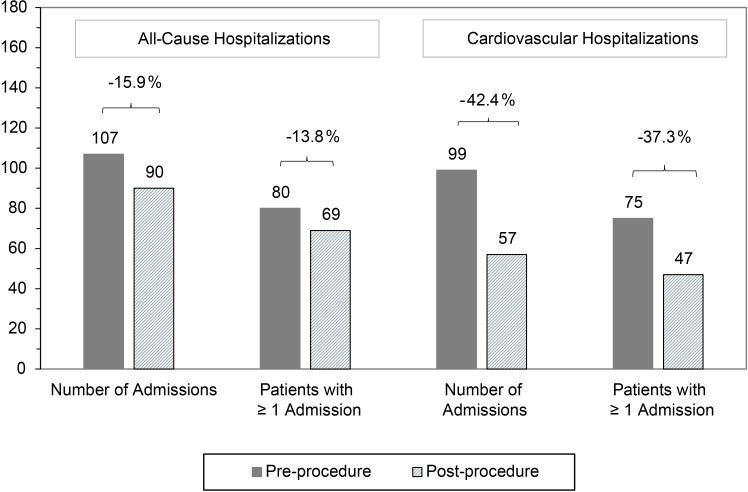
Changes in 12-month all-cause and cardiovascular hospitalisations. Reductions in cardiovascular hospitalisations are statistically significant (p=0.001 for number of admissions, p=0.004 for number of patients with admissions).

The total number of admissions and the number of patients with at least one admission was lower postprocedure for both all-cause and cardiovascular hospitalisations (figure 6). These differences were driven by the cardiovascular hospitalisations, which were reduced by 42.4% (99 to 57, p=0.001), with a similar reduction in the number of patients having a cardiovascular hospitalisation (37.3%, p=0.004). The reduction in direct current cardioversions was even greater in magnitude, with the total number decreasing by 62.3% (p<0.001) and the number of patients requiring cardioversion decreasing by 57.1% (p<0.001). Repeat ablations accounted for 61.4% of the 57 post-ablation cardiovascular hospitalisations, representing 33 patients (10.0% of the population) with 35 ablations.

## Discussion

Although some data show improvement in hard outcomes such as stroke and overall survival following AF ablation,^12 13^ the overwhelming reason for offering this treatment remains that of symptom relief. As such, it is important to systematically evaluate changes in AF-related QOL following AF ablation, and to determine factors that are likely to predict the greatest benefit. These data, along with assessment of changes in healthcare utilisation following AF ablation, should help to further improve the already documented health economic benefits from this procedure. To that end, we report multicentre study outcomes of significant reductions in healthcare utilisation and significant increases in patient-reported QOL after ablation for PAF using the standardised CLOSE protocol. We observed strong associations between the magnitude of QOL improvement and post-ablation measures of clinical success, including residual AF burden. A small but important group of patients did not experience clinically meaningful improvement in QOL, likely due to either high QOL at baseline or residual AF burden after ablation.

In addition to the impact of AF on a patient’s day-to-day activities, the impact on healthcare resource utilisation can be substantial.^14^ These findings highlight the need for treatment options that significantly reduce healthcare utilisation in AF populations. In this study, PVI ablation with the CLOSE protocol led to a 1-year reduction in cardiovascular hospitalisations of 42.4% and a 1-year reduction in cardioversions of 62.3%. As healthcare costs continue to rise, these reductions are increasingly important to both healthcare systems and patients.

Another notable aspect of this current study is the use of a broader metric of AF burden to assess the impact of ablation efficacy on QOL improvement. The most widely accepted method is to use an implantable loop recorder,^15 16^ but this involves additional invasive procedure(s) to implant and/or explant the device, and it carries the risk of false-positive diagnoses because of sensitive arrhythmia detection algorithms.^17^ Furthermore, the clinical significance of asymptomatic arrhythmias detected solely on implantable loop recorder is uncertain. Our liberal use of TTM through the 12-month follow-up period in this study allowed us to capture symptomatic arrhythmia recurrences, thereby providing a more focused assessment of clinically relevant arrhythmia burden. Freedom from any episode of AF/AFL/AT of ≥30 s duration has been recommended as the definition to use for measuring clinical success at 1 year after AF ablation, and is typically the measure reported in AF ablation studies.^18^ However, the current analysis is focused on the overall patient experience, and our results showed a strong association between residual burden and patient-reported AF-specific QOL improvement, suggesting that it is a meaningful measurement from a patient perspective. Our results are consistent with those of contemporary multicentre AF ablation studies. In CABANA, the mean overall AFEQT score in the catheter ablation group improved from 62.9 points at baseline to 86.4 points at 12 months,^19^ and in CIRCA-DOSE, the mean overall AFEQT scores in the radiofrequency group improved from 54.1 to 87.5 points at 12 months.^16^ Our study shows a similarly impressive improvement of 25.7 points in mean overall score on the AFEQT instrument, underscoring the salutary effects seen with AF catheter ablation. However, the differential impact of this procedure on individual patients’ QOL has not been studied previously. In particular, neither CABANA (Catheter Ablation vs Antiarrhythmic Drug Therapy for Atrial Fibrillation) nor CIRCA-DOSE (Cryoballoon vs Irrigated Radiofrequency Catheter Ablation: Double Short vs Standard Exposure Duration) reported on patients who derived little or no symptomatic benefit. To this end, our finding that approximately one in six patients did not achieve significant QOL improvement following AF ablation is notable. We have shown that these patients were likely to either be those who had higher levels of QOL at baseline, those with more advanced disease as reflected by higher CHA_2_DS_2_ -VASc scores or higher residual AF burden following PVI. This latter observation should spur the electrophysiology community towards striving for further improvement in ablation outcomes. Indeed, in this current study, the 12-month success rate of nearing 80% is notably higher compared with prior prospective, multicentre studies with stringent monitoring and independent core laboratory analysis such as FIRE and ICE, where the success rates were 64.1% and 65.6% in the radiofrequency and cryoballoon arms, respectively. Although a supplementary analysis of FIRE and ICE showed that healthcare utilisation rates were lower in the cryoballoon arm in spite of almost identical rates of freedom from arrhythmia,^20^ our single-arm study design did not allow such a comparison. Our observation that duration of AF history was the only variable related to freedom from arrhythmia after ablation is important, and is in line with recent publications that support early utilisation of catheter ablation.^21 22^

## Limitations

The primary limitation of the study was the single-arm design, which can make it difficult to disentangle improvements attributable to treatment from improvements due to a placebo effect. However, the magnitude of QOL improvement was shown to be significantly associated with the objective clinical measure of residual atrial tachyarrhythmia burden, suggesting that any potential placebo effect was not the primary driver of improvement in QOL. Even so, in the absence of a randomised controlled study, and given that the study was powered for efficacy and safety, but not for QOL or healthcare utilisation, our data should be considered as hypothesis-generating. An additional limitation of this study was the absence of continuous rhythm monitoring via an implantable device, which necessitated defining an alternate measure of burden. This definition could potentially be used in other studies of PAF populations, where implantable devices are typically the exception rather than the rule.

## Conclusion

Our study has demonstrated that patients with PAF with symptomatic AF experience significant increases in QOL, along with corresponding reductions in cardiovascular-related hospitalisation and cardioversion, after ablation with the standardised CLOSE PVI protocol. The magnitude of improvement in QOL was significantly associated with the level of residual AF burden. The minority of patients who did not derive significant symptomatic benefit were more likely to have had a high level of QOL at baseline, and/or were more likely to experience high residual AF burden following ablation.

**Key messages**

**What is already known on this subject?**

- Catheter ablation for paroxysmal atrial fibrillation (PAF) improves quality of life (QOL), but factors affecting magnitude of improvement in individual patients are not well described.

**What might this study add?**

- In this international multicentre study, pulmonary vein isolation with a standardised CLOSE protocol was associated with significant improvement in QOL and healthcare utilisation at 12 months.
- QOL improvement was associated with degree of impairment at baseline and inversely related to the residual AF burden after ablation.

**How might this impact on clinical practice?**

- Formal QOL assessment at baseline may help identify patients who are more likely to benefit from catheter ablation of PAF.
- Operators should continue to strive towards better ablation outcomes, as patients’ QOL improvement depends on residual arrhythmia burden.

## Data Availability

Data are available on reasonable request. Johnson & Johnson Medical Devices Companies have an agreement with the Yale Open Data Access (YODA) Project to serve as the independent review panel for evaluation of requests for clinical study reports and participant level data from investigators and physicians for scientific research that will advance medical knowledge and public health. Requests for access to the study data can be submitted through the YODA Project site at: http://yoda.yale.edu.
